# Modification of Decellularized Goat-Lung Scaffold with Chitosan/Nanohydroxyapatite Composite for Bone Tissue Engineering Applications

**DOI:** 10.1155/2013/651945

**Published:** 2013-06-13

**Authors:** Sweta K. Gupta, Amit K. Dinda, Pravin D. Potdar, Narayan C. Mishra

**Affiliations:** ^1^Department of Polymer and Process Engineering, Indian Institute of Technology Roorkee, Saharanpur Campus, Saharanpur, UP 247001, India; ^2^Department of Pathology, All India Institute of Medical Sciences, New Delhi 110029, India; ^3^Department of Molecular Medicine & Biology, Jaslok Hospital and Research Center, Mumbai, Maharashtra 400026, India

## Abstract

Decellularized goat-lung scaffold was fabricated by removing cells from cadaver goat-lung tissue, and the scaffold was modified with chitosan/nanohydroxyapatite composite for the purpose of bone tissue engineering applications. MTT assay with osteoblasts, seeded over the chitosan/nanohydroxyapatite-modified decellularized scaffold, demonstrated significantly higher cell growth as compared to the decellularized scaffold without modification. SEM analysis of cell-seeded scaffold, after incubation for 7 days, represented a good cell adhesion, and the cells spread over the chitosan/nanohydroxyapatite-modified decellularized scaffold. Expression of bone-tissue-specific osteocalcin gene in the osteoblast cells grown over the chitosan/nanohydroxyapatite-modified decellularized scaffold clearly signifies that the cells maintained their osteoblastic phenotype with the chitosan/nanohydroxyapatite-modified decellularized scaffold. Therefore, it can be concluded that the decellularized goat-lung scaffold-modified with chitosan/nanohydroxyapatite composite, may provide enhanced osteogenic potential when used as a scaffold for bone tissue engineering.

## 1. Introduction

Worldwide, over 2 million grafting procedures are performed annually for skeletal reconstruction (e.g., trauma, tumor excision, failed arthroplasty, and spinal fusion) [1], but due to inadequate availability of ideal bone substitute, the death rate has been increasing day by day. Autograft and allograft bones which are generally used for repairing bone defects result in secondary trauma and immune repulsion respectively [2]. Therefore, to solve the concerns related to current surgical skeletal reconstruction, there is a necessity to develop an ideal bone substitute or biofunctional bone which should mimic the natural bone and replace the lost/damaged bone in the human body. The biofunctional bone can be regenerated by the approach of tissue engineering, which involves 3 important elements: (i) cells, (ii) growth factors, and (iii) scaffold (a 3D porous architecture made of biomaterials, whereupon the cells are seeded) [3]. The interactions among these 3 elements (cells, scaffold, and growth factors) control the process of tissue regeneration. The design and fabrication of a perfect tissue engineering scaffold is a complex issue because the ideal scaffold should be biocompatible, biodegradable, highly porous, and so forth, and the scaffold should also intermingle with the proximate tissue to permit the scaffold to be colonized by cells. There are various methods available for scaffold fabrication, for example, gas foaming [4, 5], thermally induced phase separation [6], rapid prototyping [7], electrospinning [8, 9], solvent casting/porogen leaching, and decellularization [10]. Amongt these, decellularization is one of the most promising methods for scaffold fabrication, because it generates scaffold from bioorigin (e.g., cadaver tissue in this study), which consists of various types of biopolymers required for tissue regeneration. Besides, this method is simple, economical and provides a scaffold that retains the original architecture of tissue/extracellular matrix (ECM). Decellularization is a method where cells are completely removed from the cadaver organ/tissue without adversely affecting its 3D ultrastructure of the tissue skeleton [10]. Decellularization technique has already been exploited for fabricating scaffold from various xenogenic tissues/organs (e.g., urinary bladder [11], small intestinal submucosa [12], heart valves [13], pericardium [14], blood vessels [15], nerves [16], skeletal muscle [17], tendon [18], ligament [19], liver [20], and skin [21]) of rat, pig, buffalo, cattle, and so forth, but, *to the best of our knowledge, goat-lung tissue has not yet been applied for decellularization to fabricate scaffold for tissue engineering applications*. Here, *our aim is to fabricate scaffold from cadaver goat-lung tissue by decellularization and to modify it for bone tissue regeneration.* There might be some important signalling/growth factors embedded within the decellularized goat-lung scaffold which can provide physicochemical cues needed to guide the cellular development.

Natural bone tissue consists of organic-inorganic nanocomposite in which hydroxyapatite (HA, Ca_10_(PO_4_)_6_(OH)_2_) crystals are deposited on fibrous collagen [22]. The hybrid of chitosan (organic) and hydroxyapatite (inorganic) composite scaffolds has received great attention for bone tissue engineering applications, because they can mimic the natural bone composition [23]. Therefore, from the biomimetic point of view, before applying goat-lung scaffold for bone tissue regeneration, we aim to modify the decellularized goat-lung scaffold with chitosan/nanohydroxyapatite composite and evaluate the modified (with chitosan/nanohydroxyapatite composite) decellularized scaffold for bone tissue engineering applications.

## 2. Materials and Methods

### 2.1. Materials

All the chemicals employed in this work were of analytical grade and purchased from different firms: Sigma Aldrich, Himedia, and Rankem. Chitosan (CS) from crab shells (C_12_H_24_N_2_O_9_, deacetylation degree ≥75%) was purchased from Sigma Aldrich Inc., USA. Hydroxyapatite (calcium phosphate-tribasic-nanopowder, <200 nm particle size, ≥97% synthetic) was supplied by Sigma. Hydroxyapatite nanopowder will be called nHAp in this paper. Ammonia solution (NH_4_OH, molecular weight 35.05 g/mol) was purchased from Rankem. Autoclaved double-distilled water was used throughout the experiment.

### 2.2. Fabrication and Characterization of Decellularized Scaffold from Goat-Lung Tissue

The decellularization of goat-lung tissue was done by enzymatic method using trypsin. In brief, the goat-lung tissue was sectioned and treated with 0.25% Trypsin-EDTA in 1X PBS (Dulbecco's phosphate-buffered saline, Himedia, India) supplemented with 1% Pen-Strep solution (Himedia, India) for 24 h at 37°C. They were then further treated with 0.1% SDS in 1X PBS at 37°C for 6–8 h, followed by agitating in solution containing RNase (20 *μ*g/mL, Himedia, India) and DNase (0.2 mg/mL, Himedia, India) for 24 h at 37°C, followed by intensive wash cycles with 1X PBS. The goat-lung decellularized scaffolds were disinfected by agitating in an aqueous solution of 0.1% (v/v) hydrogen peroxide and 4% (v/v) ethanol. The decellularity of the fabricated scaffold was assessed by DNA quantification and hematoxylin and eosin staining. The decellularized scaffold was then morphologically characterized by SEM (scanning electron microscopy), and chemical analysis of scaffold samples was performed with LEO 1525 scanning electron microscope equipped with an EDS (energy dispersive spectroscopy) detector (Carl Zeiss SMT Ltd., Hertfordshire, UK). To quantify the chemical content of small areas (~60 *μ*m^2^) on the sample surface, distinctly different topographical areas were chosen from the sample and examined at 10 kV using INCA Energy 3000 software.

### 2.3. Preparation and Characterization of CS/nHAp Composite Particles

The coprecipitation method has been widely used to prepare composite material involving nHAp [24]. Here, in this study, nHAp has been mixed with chitosan, by using coprecipitation technique, to develop CS/nHAp composite. For preparing CS/nHAp composite particles, a clear and homogenous solution of chitosan was made in dilute acetic acid. Chitosan (1 wt%) solution was prepared by dissolving 1 g of chitosan in 100 mL of 1% (v/v) acetic acid and stirring overnight. Simultaneously, 1 g of nanohydroxyapatite powder (nHAp) was accurately weighed and mixed with 100 mL of autoclaved double-distilled water to form the suspension. The nHAp suspension was then added to the chitosan solution in the ratio of 4 : 6 wt% (CS : nHA) under vigorous stirring to obtain a uniform dispersion of hydroxyapatite in the solution mixture. The solution mixture was then stirred at 45°C for 10 min. This was followed by addition of NH_4_OH dropwise into the dispersed mixture with vigorous stirring. Eventually the solution turned opalescent, suggesting the formation of CS/nHAp composite particles. Finally, the precipitate was filtered, and the excess acetic acid and ammonia were removed from the resultant mixture through dialysis. The CS/nHAp composite particle, so formed, was characterized by FTIR (Fourier transform infrared spectroscopy) and AFM (atomic force microscopy). FTIR for CS, nHAp, and CS/nHAp composites was carried out using Nexus Thermo spectrophotometer (ThermoNicolet, USA) over a range between 4000 and 400 cm^−1^ at 2 cm^−1^ resolution, averaging 100 scans. Samples were processed to form compressed potassium bromide disks. Samples were taken in the ratio of 1 mg per 900 mg potassium bromide. AFM was also performed to find CS/nHAp composite's particle size and its morphology.

### 2.4. Modification of Decellularized Scaffold with CS/nHAp Composite and Its Characterization

Decellularized goat-lung tissue was modified with CS/nHAp composite to form the CS/nHAp-modified decellularized scaffold. Modification of decellularized scaffold was achieved by chemically attaching the decellularized scaffold and CS/nHAp composite with the help of EDC (1-ethyl-3-[3-dimethylaminopropyl] carbodiimide) and NHS (N-hydroxysuccinimide). The crosslinking solution contains 1X PBS (pH 7.4) with a final concentration of 2 mM EDC and 5 mM NHS. The decellularized tissues were reacted with this modified/crosslinking solution at room temperature for 15 min to activate the carboxyl groups present on the collagen molecules of the decellularized goat-lung tissue (collagen-based biomaterial) [25]. After incubation, the CS/nHAp composite was added to the crosslinking solution containing decellularized scaffold. The incubation continues for 24 h at room temperature on an orbital shaker table at 80–100 rpm. Following incubation, the resultant crosslinked tissues were rinsed with 1X PBS for 2 h on an orbital shaker table with several changes of 1X PBS solution to remove residual crosslinkers and unbound CS/nHAp composite particles. The CS/nHAp-modified decellularized scaffold was then characterized by EDS and FTIR.

The decellularized goat-lung scaffolds were modified/dipcoated with chitosan and nanohydroxyapatite by immersing in a solution of chitosan (1 wt%) in acetic acid (1% v/v) and nHA (1 wt%) in distilled water, respectively, for 5 h at 37°C. The chitosan-modified decellularized scaffold and nanohydroxyapatite-modified decellularized scaffold were then rinsed with 1X PBS to remove residual chitosan and nanohydroxyapatite before use.

### 2.5. Human Osteoblast Cell Culture

Human osteoblast cells were obtained from Jaslok Hospital and Research Center, Mumbai, India. The osteoblast cells were cultured in Dulbecco's modified Eagle's medium (DMEM, Himedia, India) supplemented with 10% fetal bovine serum (FBS, GIBCO), 1% penicillin-streptomycin solution (Himedia, India), 1 *μ*L/mL Insulin (Sigma, USA), and 2 *μ*L/mL glutamine (Himedia, India). Osteoblast cells were grown as monolayer cultures in T-75 flasks (Nunc, India) and were subcultured twice a week at 37°C in an atmosphere containing 5% CO_2_ in air and >95% relative humidity.

### 2.6. Evaluation of *In Vitro* Biocompatibility Using Human Osteoblast Cells

Human osteoblast cell behaviors with decellularized goat-lung scaffold, chitosan-modified decellularized scaffold, nHAp-modified decellularized scaffold, and CS/nHAp-modified decellularized scaffold were individually determined by MTT (3-[4,5-dimethylthiazol-2-yl]-2,5- diphenyltetrazolium bromide) assay. Briefly, all the scaffolds (1 mm × 1 mm pieces) were plated, in triplicate, in a 96-well culture plate to recondition them by incubating in DMEM overnight at 37°C in CO_2_ incubator. The next day, osteoblast cells were released from the flask by trypsinization and resuspended at a density of 5 × 10^3^ cells per well, in triplicate, and incubated for 7 days at 37°C in CO_2_ incubator. After a respective time period of 7 days, 90 *μ*L of fresh complete DMEM media and 10 *μ*L of MTT solution (5 mg/mL stock in PBS) were added to each well (final volume of 100 *μ*L). The plate was incubated at 37°C for 4 h until purple formazan crystals were formed. The medium was then discarded and 200 *μ*L of DMSO (dimethyl sulphoxide) was added to the wells and mixed properly to dissolve the formazan crystals. The plate was then swirled gently. Absorbance values were blanked against DMSO, and the absorbance of cells exposed to medium only (i.e., no scaffold added) was taken as control. The absorbance was read at 490 nm using a Microplate Reader Model Sunrise (TECAN, India) with the subtraction of plate absorbance at 650 nm. The absorbance was plotted against the number of days.

### 2.7. Histological Examination of the CS/nHAp-Modified Recellularized Scaffold

Osteoblast cells were seeded onto the CS/nHAp-modified decellularized scaffold and were maintained at 37°C under 5% CO_2_ in a humidified atmosphere. CS/nHAp-modified recellularized scaffolds were collected after 7 days, in duplicate, washed in 1X PBS, and fixed in 2.5% glutaraldehyde at room temperature. The samples were then embedded in paraffin and longitudinally cut into 5 *μ*m thick sections. The sections were mounted on slides and stained with hematoxylin and eosin staining to identify the cellular components present in the CS/nHAp-modified recellularized scaffold.

### 2.8. Morphological Examination of the CS/nHAp-Modified Recellularized Scaffold after a 7-Day Cell Attachment Study

The human osteoblast cells were seeded onto the CS/nHAp-modified decellularized scaffold and incubated for 7 days. The recellularized scaffolds, after 7 days, were harvested and fixed in 2.5% glutaraldehyde at room temperature. The samples were then washed in 1X PBS and observed under SEM at the saturation pressure of water vapour (1 torr) and an accelerating voltage of 15 kV. Images showing surface morphologies of osteoblast cells-CS/nHAp-modified decellularized scaffold constructs were taken at 250x, 500x, and 1000x magnifications.

### 2.9. Gene Expression Study

The *in vitro *cellular response of human osteoblast cells to the CS/nHAp-modified decellularized scaffold has been demonstrated by gene expression studies. Molecular expression of mesenchymal stem cell marker (CD105, CD73, CD44), pluripotency (Nanog), differentiation (Lif), and bone-tissue-specific marker (OCN), which are responsible for repair and regeneration process of bone, was carried out for osteoblast cells in presence of CS/nHAp-modified decellularized scaffold, for 7 days, to determine the differences in transcriptional response of cells. OCN is secreted solely by the osteoblast cells [26], and therefore, was used as a tissue-specific biomarker in bone formation, bone mineralization, and calcium ion homeostasis [27, 28]. *β*-Actin marker (housekeeping gene) was used as internal control.

### 2.10. Statistical Analysis

Cell viability and cell proliferation experiments were performed in triplicate (*n* = 3), and the experimental results are represented as mean values ± standard deviations (SD). Using MINITAB statistical software (MINITAB Release 13.32), statistical analysis (one-way ANOVA test) was performed with *P* < 0.05 considered as being statistically significant.

## 3. Results

### 3.1. Fabrication and Characterization of Decellularized Scaffold from Goat-Lung Tissue

**Figure 1:**
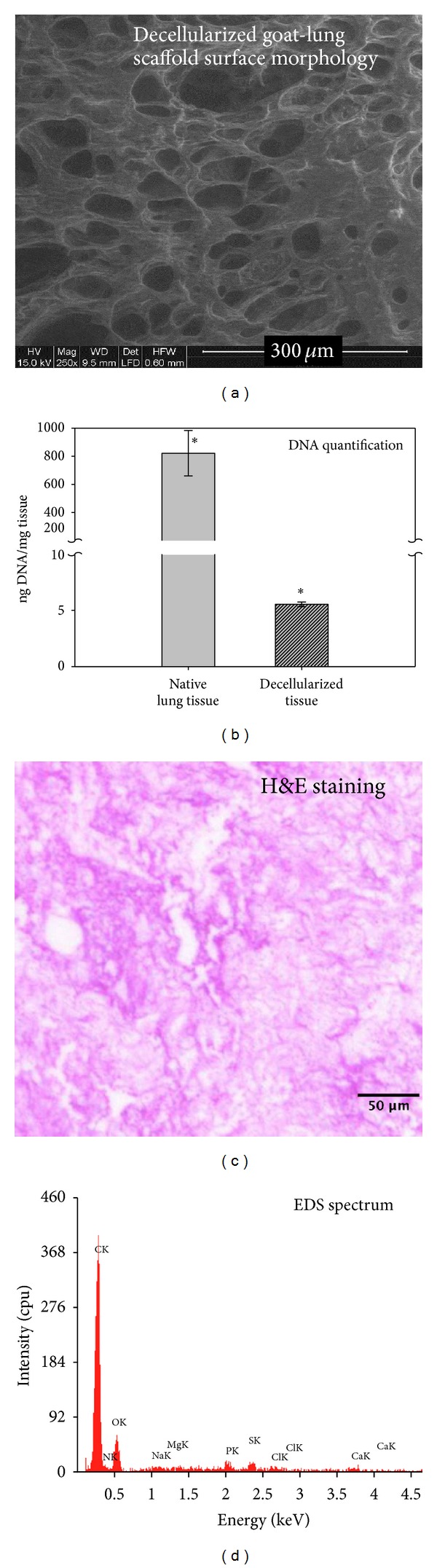
(a) Scanning electron micrograph of decellularized goat-lung tissue at 250X showing porous architecture. (b) DNA content in native lung tissue and the decellularized tissue. Native tissue served as a positive control. (c) Hematoxylin and eosin staining of decellularized lung tissue section showing collagenous protein structure with negative cell nuclei staining. No nuclear counterstaining was detectable, indicating a total cell removal. (d) EDS spectrum of the decellularized scaffold, showing carbon and oxygen as the major constituents of scaffold, confirming its organic nature. (*) stands for significant difference between native lung tissue and decellularized tissue (*P* < 0.05).

Goat-lung scaffold was fabricated by decellularizing cadaver goat lung by employing trypsin-SDS-based decellularization method as discussed in Section 2.2. The scaffold was characterized by SEM, DNA quantification, H&E staining, and EDS (Figure 1). Morphological examination of the surface of decellularized scaffold, by SEM, confirms the porous nature of the goat-lung tissue scaffold, as expected for a porous lung (Figure 1(a)). The DNA quantification assay indicated significant removal of the cellular material from the native goat-lung tissue (Figure 1(b)). H&E staining of decellularized-scaffold section shows collagenous ECM structure with negative cell nuclei staining (Figure 1(c)), which indicates the nonexistence of any cell in the scaffold. The EDS spectrum (Figure 1(d)) of the decellularized scaffold indicates that the tissue contains mainly carbon and oxygen with small amount of sodium, chlorine, magnesium, and sulphur. The presence of carbon and oxygen elements indicated the organic nature of the goat-lung tissue, and therefore, it is predicted to be biodegradable inside the body.

### 3.2. Characterization of CS/nHAp Composite Particles

**Figure 2:**
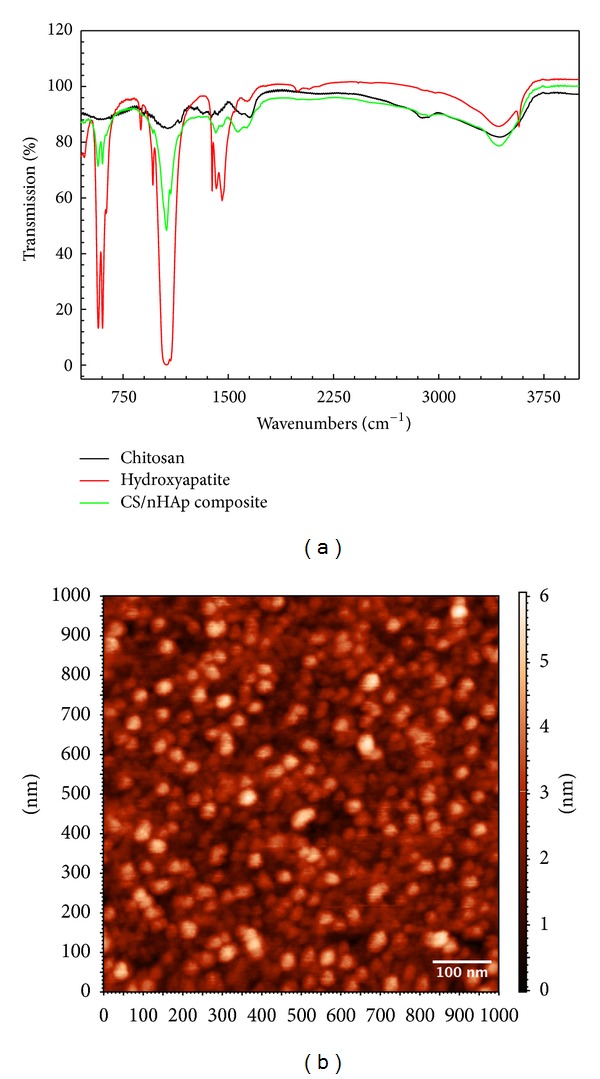
(a) FTIR spectrum of chitosan, hydroxyapatite, and CS/nHAp composite. (b) AFM image of CS/nHAp composite particles.

CS/nHAp composite was characterized by comparing the absorption bands arising from the IR spectrum of nHAp and CS standard sample. The FTIR spectrum of chitosan (Figure 2(a)) shows a broad –OH (3450–3100 cm^−1^) and aliphatic C–H (2990–2850 cm^−1^) stretching bands. Another major absorption band between 1220 and 1020 cm^−1^ represents the free primary amino group (–NH_2_) at C_2_ position of glucosamine, a major group present in chitosan. The band at 1647 cm^−1^ represents acetylated amino group of chitin, which indicates that the sample is not fully deacetylated. In the FTIR spectra of nHAp (Figure 2(a)), the broader band at 1000–1040 cm^−1^ represents the stretching vibration of P–O. The two absorption bands, which appeared at 603 cm^−1^ and 566 cm^−1^, correspond to the bending vibration of P–O. The absorption bands at 3425 cm^−1^ and 1641 cm^−1^ correspond to the stretching and bending vibration of H–O, respectively. The band at 1422 cm^−1^ and 874 cm^−1^ is stretching and bending vibration of C=O, respectively. The FTIR spectrum of CS/nHAp composite (Figure 2(a)) contains the entire characteristic absorption bands of CS and nHAp. In comparison to chitosan, CS/nHAp composite is characterized by two absorption bands at 635 cm^−1^ and 955 cm^−1^, corresponding to stretching vibration bands of P–O from PO_4_^3−^ and bending deformation mode of O–H from nHAp, confirming the successful formation of CS/nHAp composite scaffold [29]. AFM study (Figure 2(b)) demonstrated spherical shape of CS/nHAp composite particles with size in the range of 61–97 nm diameters.

### 3.3. Modification of Decellularized Goat-Lung Scaffold with CS/nHAp Composite and Its Characterization

Decellularized goat-lung scaffold was modified/crosslinked with CS/nHAp composite using EDC and NHS. In this study, EDC and NHS were added to increase the rate and amount of crosslinking between –COOH groups present in the decellularized goat-lung tissue (collagen-based biomaterial) and –NH_2_ groups present in the CS/nHAp composite, thereby increasing the compressive stiffness of the scaffold [30–32].

#### 3.3.1. Characterization of CS/nHAp-Modified Decellularized Scaffold by EDS and FTIR

**Figure 3:**
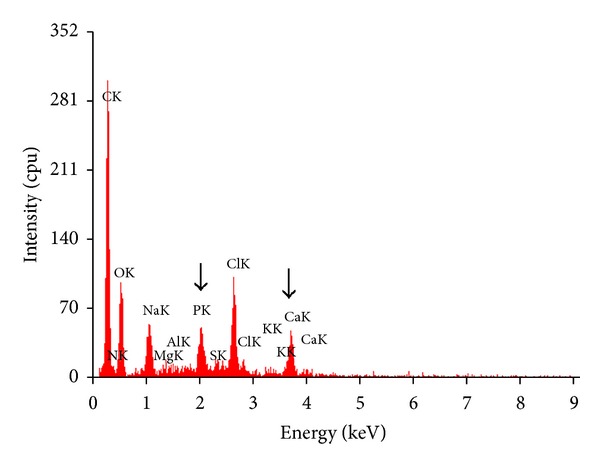
EDS spectrum of the CS/nHAp modified decellularized scaffold showing the presence of Phosphorous and Calcium peaks due to the presence of nHAp composite in the scaffold. Arrows represent the Phosphorous and Calcium peaks in the CS/nHAp modified decellularized scaffold.

To confirm the modification of the decellularized scaffold, CS/nHAp-modified decellularized scaffold was characterized by energy dispersive spectroscopy (EDS), which demonstrated the presence of phosphate (P) and calcium (Ca) peaks within the CS/nHAp-modified decellularized scaffold (Figure 3), whereas, the respective peak of P and Ca was absent in the EDS spectrum (shown in the Figure 1(d)) of decellularized goat-lung scaffold. This result suggested that the decellularized scaffold was modified by CS/nHAp composite. Peaks corresponding to Sodium (Na) and chlorine (Cl) might be present in the spectrum because the scaffolds were stored in saline solution. As the CS/nHAp-modified decellularized scaffold was made of natural materials (calcium, phosphorous, and collagen) found in bone tissue, it makes an excellent biocompatible/biodegradable material for bone tissue regeneration.

**Figure 4:**
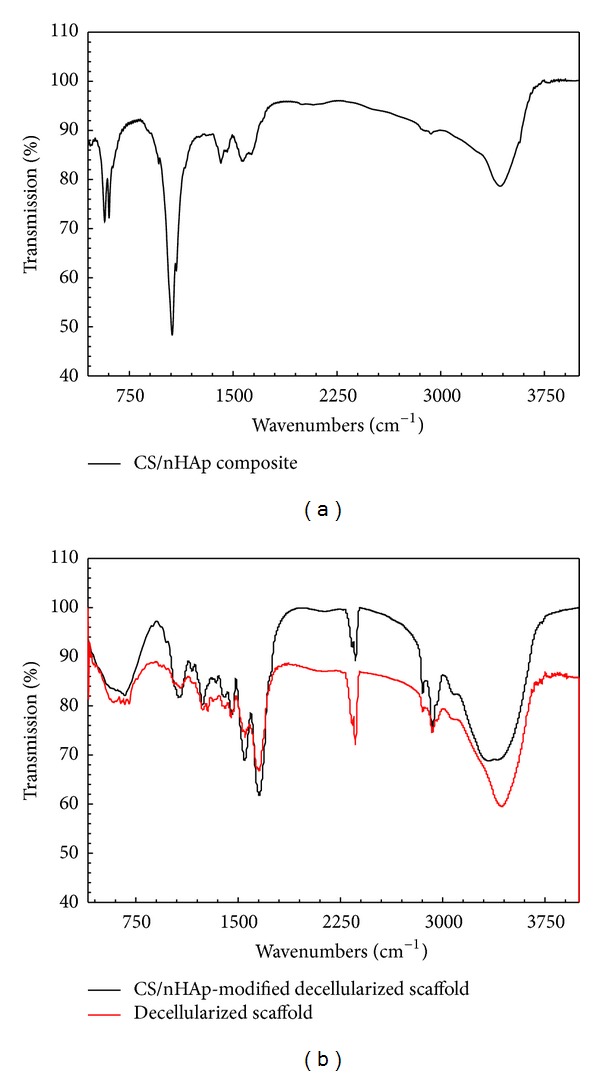
FTIR spectra of (a) CS/nHAp composite and (b) CS/nHAp-modified decellularized scaffold and decellularized scaffold. Peak corresponding to the free amino group (between 1220 and 1020 cm^−1^) is present in the CS/nHAp composite, but got reduced in the CS/nHAp-modified decellularized scaffold, indicating the crosslinking of –NH_2_ group of CS/nHAp composite with the –COOH group of the decellularized scaffold.

FTIR analysis has also been performed as a complementary study to characterize the CS/nHAp-modified decellularized scaffold formation. If we compare the FTIR of CS/nHAp composite and CS/nHAp-modified decellularized scaffold, it is evident that the peak intensity of free amino group (–NH_2_) at C_2_ position of glucosamine (1220–1020 cm^−1^) for CS/nHAp composite has got significantly reduced in the IR spectra of CS/nHAp-modified decellularized scaffold (Figure 4). The IR spectrum of CS/nHAp-modified decellularized scaffold and decellularized scaffold (Figure 4(b)) does not exhibit much change, which leads to the conclusion that crosslinking of CS/nHAp composite does not disturb the original matrix structure of the decellularized scaffold. 

### 3.4. Osteoblast Cell Behavior on the Scaffold

#### 3.4.1. Cell Viability, Proliferation, and Attachment over the Scaffold

**Figure 5:**
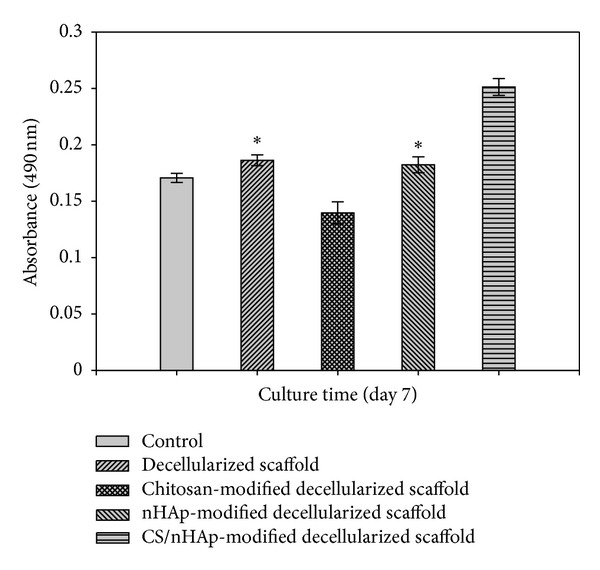
Osteoblast cell viability and proliferation assessed by MTT assay for 7 days. Absorbance was measured in replicates of three and the calculated standard error of the mean (SEM) plotted as error bars. Here, absorbance (490 nm) is directly proportional to cell viability. Osteoblast cell proliferation with CS/nHAp modified decellularized scaffold is shown to be highest after the 7 days of incubation. (*) stands for insignificant difference (*P* > 0.05) between the scaffolds. (*) unmarked bars show significant difference between the scaffolds (*P* < 0.05).

MTT assay was performed to assess the osteoblast cell viability and proliferation over goat-lung decellularized scaffold, chitosan-modified decellularized scaffold, nHAp-modified decellularized scaffold, and CS/nHAp-modified decellularized scaffold. Osteoblast cells seeded on a tissue culture plate without scaffold were taken as control. The absorbance value, that is, O.D., a measure of cell viability, was determined for the cell-seeded scaffolds. The result showed that osteoblast cells were viable in all the scaffolds, but a significant increase was observed in the cell viability of osteoblasts with CS/nHAp-modified decellularized scaffold as compared to the other scaffolds tested (Figure 5). Though there was no significant difference in the proliferation of osteoblast cells in decellularized scaffold and nHAp-modified decellularized scaffold, chitosan-modified decellularized scaffold shows less osteoblast cell proliferation because chitosan lacks bone-bonding bioactivity [24]. This result is also supported by a study that demonstrates that osteoblasts exhibit significantly higher proliferation capacity on the CS/nHAp scaffold as compared to that on pure chitosan scaffolds [29].

**Figure 6:**
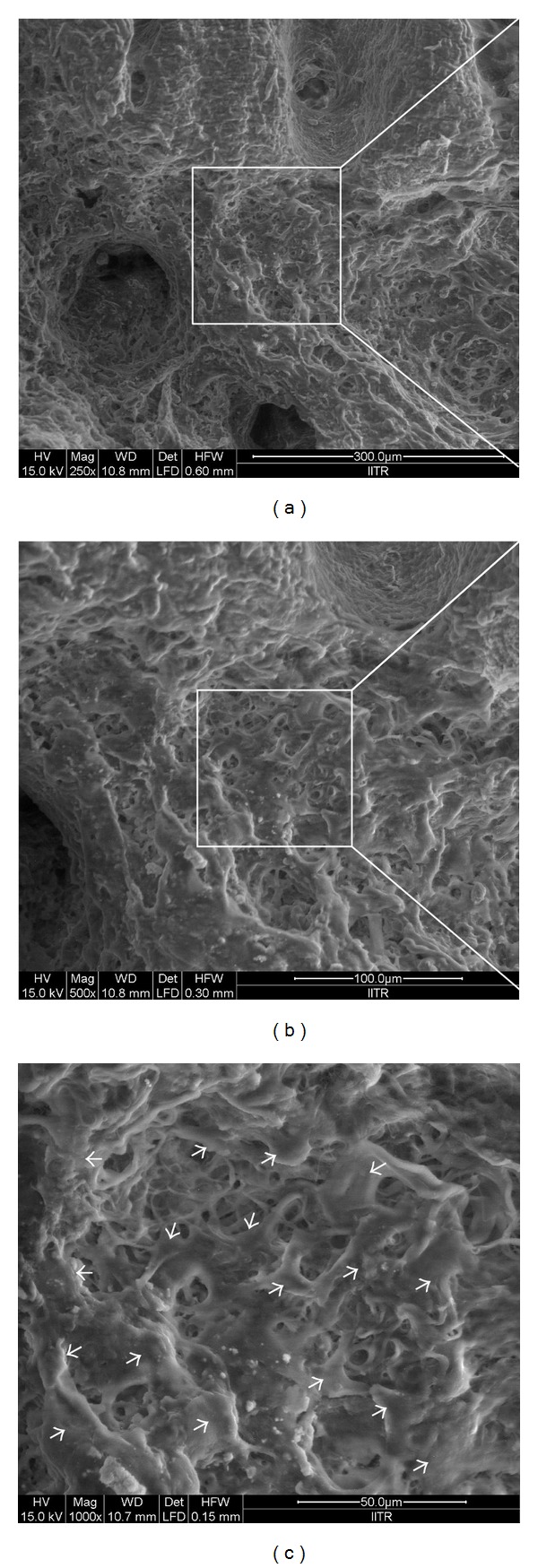
SEM images of the recellularized CS/nHAp-modified decellularized scaffold after 7 days of osteoblast cell seeding at 250x (a), 500x (b), and 1000x (c) magnifications. Arrows represent the osteoblast cells over the CS/nHAp-modified decellularized scaffold.

Figure 6 shows the scanning electron microscopic image of osteoblast cell adhesion and cell interaction with CS/nHAp-modified decellularized scaffold. After 7 days of incubation, it was observed that cells get adhered to the scaffold and have polygonal morphology with the cell membrane being somewhat flattened onto the rough surface of the CS/nHAp-modified decellularized scaffold, created by CS/nHAp particles. SEM image of osteoblast cell-scaffold (CS/nHAp-modified decellularized scaffold) construct also depicts that osteoblast cells have spread all around the porous structure of the scaffold, and it becomes more clear when compared to control image (decellularized scaffold without cells, Figure 1(a)). This result was also supported with MTT assay, which confirmed the potential of CS/nHAp-modified decellularized scaffold towards bone tissue regeneration.

#### 3.4.2. Histological Examination of the Recellularized CS/nHAp-Modified Scaffold

**Figure 7:**
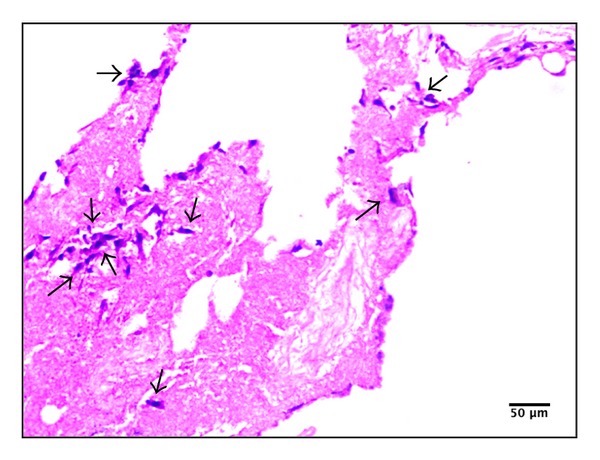
Hematoxylin and eosin staining of osteoblast recellularized CS/nHAp-modified scaffold. Images at magnification 10x visualize the scaffold matrix after a successful 7-day recellularization of osteoblast cells. Nuclear counterstaining was detectable, identifying the cells growing inside the CS/nHAp-modified scaffold. Hematoxylin stains the nuclei of osteoblast cells blue, and the eosin stains the scaffold ECM structures pink. Arrows represent the osteoblast cells within the CS/nHAp-modified scaffold.

Figure 7 illustrates H&E staining image of CS/nHAp-modified recellularized scaffold incubated with the osteoblast cells for a period of 7 days. H&E staining shows the presence of osteoblast cells in the CS/nHAp-modified recellularized scaffold. The nuclear counterstaining of multiple cellular groups of osteoblast cells (stained blue) surrounded by collagenous protein structure (stained pink) was detectable in the CS/nHAp-modified recellularized scaffold; this indicates that the osteoblast cells might have got infiltrated through the surface of the scaffold to the interior side, as expected from a highly porous scaffold. The result of H&E staining further supports the growth and proliferation of osteoblast cells over CS/nHAp-modified decellularized scaffold, thereby proving great prospective of this scaffold for bone tissue engineering applications.

#### 3.4.3. Gene Expression Study: Molecular Marker Expressions in Cell-Scaffold Construct

**Figure 8:**
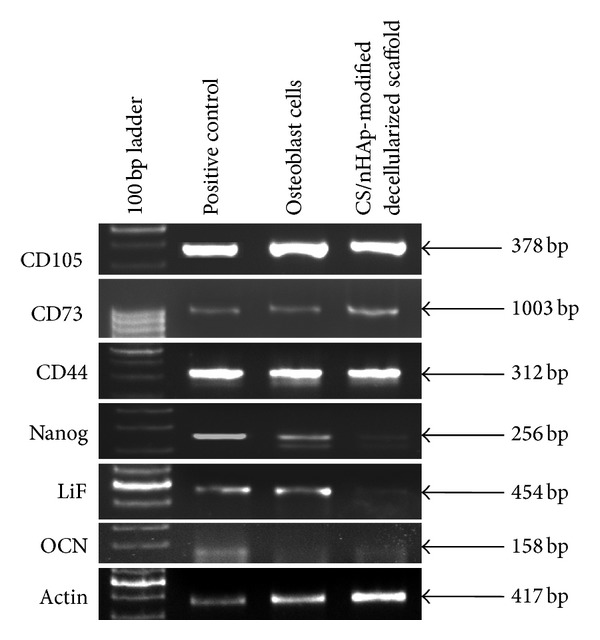
Gene expression profile of osteoblast cells after incubating with CS/nHAp-modified decellularized scaffold for 7 days. Here, actin has been used as a housekeeping gene (internal control).

Gene expression profiling of CD105, CD73, CD44, Nanog, Lif, and OCN has been demonstrated for *in vitro *cellular responses of human osteoblast cells to the CS/nHAp-modified decellularized scaffold (Figure 8). CD105, CD73, and CD44 genes are found to be expressed in the osteoblast cells seeded over CS/nHAp-modified decellularized scaffold. The expression of mesenchymal stem cell markers (CD105, CD73, and CD44) in the osteoblast cells does not exhibit any significant change in expression, which indicates that cells are viable and proliferating inside the CS/nHAp-modified scaffold. It was found that Nanog and Lif genes were not expressed in the osteoblast cells seeded over CS/nHAp-modified scaffold. Osteocalcin (OCN), a noncollagenous protein, is responsible for calcium ion binding and is a marker of bone mineralization [33]. OCN was expressed in the osteoblast cells seeded over CS/nHAp-modified scaffold.

## 4. Discussion

In this paper, we evaluated the suitability of the decellularized goat-lung scaffold modified with CS/nHAp composite for bone tissue engineering applications. We employed trypsin-SDS-based decellularization method for removing the cellular components from the goat-lung tissues. The results of SEM (Figure 1(a)) of decellularized goat-lung scaffold indicated cell removal, good porosity, and pore-to-pore interconnectivity within the scaffold. The porous nature of the goat-lung tissue scaffold was expected for the scaffold as it was derived from a highly porous cadaver lung. DNA quantification assay and H&E staining of decellularized goat-lung scaffold (Figures 1(b) and 1(c)) confirmed the significant removal of cellular components from the native goat-lung tissues, demonstrating the use of decellularized goat-lung scaffold as a template for the growth and proliferation of osteoblast cells. The EDS spectrum of decellularized goat-lung tissue (Figure 1(d)) indicated carbon and oxygen as the major components, which verifies that the scaffold will be gradually degraded in the body after implantation. 

Chitosan and nHAp have been studied extensively and used as a scaffold/biomaterial for bone tissue engineering applications [23]. A composite biomaterial CS/nHAp is expected to show an increased osteoconductivity and biodegradation together with sufficient mechanical strength for orthopedic use due to the synergistic effect of chitosan and hydroxyapatite [34]. Therefore, the decellularized goat-lung scaffold was modified/crosslinked with CS/nHAp composite for making the scaffold suitable for bone tissue regeneration. 

A composite of CS and nHAp was synthesized and characterized for studying the interaction between CS and nHAp molecules. The FTIR spectrum of CS/nHAp composite (Figure 2(a)) demonstrated no shifting of peaks of any group in the composite spectrum and no new peak formation, which shows that CS/nHAp composite is only a mixture and that no chemical reaction had taken place between CS and nHAp during the composite formation. This indicates that the original characteristics of CS and nHAp were not lost, and the CS/nHAp composite, as a whole, will be an effective osteoinductive material. The AFM study (Figure 2(b)) indicated that the CS/nHAp composite particles were in the nanometer range (67–91 nm diameter), which will provide more surface area for osteoblast cells for adhesion and proliferation.

**Figure 9:**
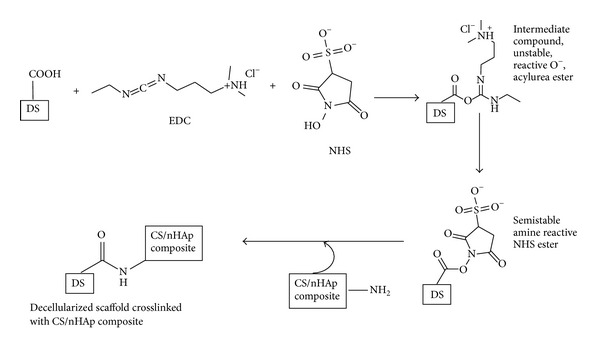
Schematic representing possible crosslinking reaction between decellularized scaffold and CS/nHAp composite in the presence of EDC and NHS. Nucleophilic attack by NH_2_ group of chitosan leads to formation of amide bond between COOH group of decellularized scaffold and NH_2_ group of CS/nHAp composite. EDC and NHS catalyze the covalent bindings between carboxylic acid and amino groups, thus, crosslinking CS/nHAp composite with the decellularized scaffold and forming CS/nHAp-modified decellularized scaffold. DS: decellularized scaffold, EDC: 1-ethyl-3-[3-dimethylaminopropyl] carbodiimide, NHS: N-hydroxy succinimide.

 Decellularized goat-lung scaffold was modified/crosslinked with CS/nHAp composite using EDC and NHS.  Figure 9 explains the possible interaction between –COOH groups present in the decellularized goat-lung tissue (collagen-based biomaterial) and –NH_2_ groups present in the CS/nHAp composite. The carboxyl groups of decellularized tissues are activated to give O-acylurea ester groups which ultimately crosslink with free amine groups of CS/nHAp composite (Figure 9). EDC and NHS helped in accelerating the crosslinking reaction.

The EDS spectrum of CS/nHAp-treated decellularized scaffold (Figure 3) result indicated that the decellularized scaffold was modified by CS/nHAp composite. Peaks corresponding to sodium (Na) and chlorine (Cl) might be present because the scaffolds were stored in saline solution. 

The FTIR analysis of CS/nHAp-modified decellularized scaffold (Figure 4) showed a significant reduction in the free amino group (–NH_2_) when compared with CS/nHAp composite, which might be due to crosslinking of (–NH_2_) groups of CS/nHAp composites with the (–COOH) groups of the decellularized scaffold (Figure 9) during the formation of CS/nHAp-modified decellularized scaffold. The EDS spectrum (Figure 3) of modified decellularized scaffold (with CS/nHAp composite) shows high oxygen content along with adequate mineralization of CS/nHAp-modified decellularized scaffold, which can help in maintaining the necessary bone mineral density and balance for the loss of oxygen transported to the osteoblast cells. Moreover, as the CS/nHAp-modified decellularized scaffold was made of natural materials (calcium, phosphorous, and collagen) found in bone tissue, it makes an excellent biocompatible/biodegradable material for bone tissue regeneration. 

In the cell behavior study, MTT assay (Figure 5) shows that all the scaffolds (decellularized scaffold, chitosan-modified decellularized scaffold, nHAp-modified decellularized scaffold, and CS/nHAp-modified decellularized scaffold) supported the growth and proliferation of osteoblast cells. However, osteoblast cells exhibited higher proliferative tendency over CS/nHAp-modified decellularized scaffold. Osteoblasts are highly sensitive to chemical-physical properties of the scaffolds. The better proliferation of osteoblasts over CS/nHAp-modified decellularized scaffold is believed to be due to higher attachment of osteoblasts, which might be due to the presence of chitosan, nHAp, and ECM of the decellularized scaffold. The synergistic effect of chitosan and nHAp [34] in the CS/nHAp-modified decellularized scaffold might have provided bone-like microenvironment to the osteoblast cells, which may also lead to an increase in osteoblast activity, including cell growth and proliferation. Surface composition (chitosan and nHAp) and roughness provided by CS/nHAp particle in the CS/nHAp-modified decellularized scaffold might have provided necessary chemical cues to the osteoblast cells, which are influenced in higher cell proliferation. Though there was no significant difference in the proliferation of osteoblast cells in decellularized scaffold and nHAp-modified decellularized scaffold, chitosan-modified decellularized scaffold shows less osteoblast cell proliferation because chitosan lacks bone-bonding bioactivity [24]. This result is also supported by a study that demonstrates that osteoblasts exhibit significantly higher proliferation capacity on the chitosan/hydroxyapatite scaffold as compared to that on pure chitosan scaffolds [29]. When the surface of the CS/nHAp-modified osteoblast recellularized scaffold was analyzed by SEM, osteoblast polygonal morphology with the cell membrane being somewhat flattened onto the rough surface (created by CS/nHAp particles) of the CS/nHAp-modified decellularized scaffold, was observed (Figure 6). This type of morphology of osteoblast cells was reported previously on the collagen-hydroxyapatite composite scaffold for bone tissue engineering [35]. The CS/nHAp particles, with their rough surface and nanofeatures, are believed to be the main enhancing factor for good cell attachment and well-spread morphology of the osteoblast cells over CS/nHAp-modified decellularized scaffold. H&E staining of CS/nHAp-modified osteoblast recellularized scaffold (Figure 7) proved that osteoblast cells not only get adhered to the surface of the decellularized goat-lung tissue but are also infiltrated through the surface of the decellularized scaffold to the interior side, as expected from a highly porous scaffold. The gene expression study (Figure 8) demonstrated that the osteoblast cells maintained their phenotype after getting incubated for 7 days *in vitro* with the CS/nHAp-modified decellularized scaffold. The absence of the expression of Nanog and Lif genes in the osteoblast cells with CS/nHAp-modified scaffold indicates the nonpluripotency of the osteoblast cells in presence of the scaffold. This may be due to the combined effect of CS, nHAp, and ECM of scaffold. Osteocalcin (OCN), a non-collagenous protein, is responsible for calcium ion binding and is a marker of bone mineralization [33]. OCN was expressed in the osteoblast cells seeded over CS/nHAp-modified scaffold, which indicates the potentiality of the scaffold to act as osteoblast cell carrier for bone tissue engineering application. It also signifies that the cells maintained their osteoblastic phenotype within the CS/nHAp-modified decellularized goat-lung scaffold, which can be applied for bone tissue regeneration.

The present study underscores that the modification of decellularized goat-lung scaffold with CS/nHAp composite has the potential to act as osteoblast cell carrier and favors the growth and proliferation of osteoblast cells, thereby, proving great prospective of this scaffold for bone tissue engineering applications.

## 5. Conclusions

Goat-lung tissue was successfully decellularized and modified with CS/nHAp composite for bone tissue engineering application. The CS/nHAp-modified decellularized scaffold is biocompatible and forms a favorable three-dimensional matrix that leads to improved osteoblast activity including cell adhesion, growth, and proliferation. The good proliferation of the osteoblasts over the CS/nHAp-modified decellularized scaffold is believed to be due to the higher attachment of cells over the scaffold. The higher attachment might be due to the presence of nHAp particles (inorganic cues) and the roughness provided by nHAp particles along with chitosan, which positively affected the growth and proliferation of osteoblasts. The osteoblast cells maintained their osteoblastic phenotype within the CS/nHAp-modified decellularized goat-lung scaffold. Thus, CS/nHAp-modified decellularized goat-lung scaffold can be applied for bone tissue regeneration.
